# Supplementary material for: The interactome of intact mitochondria by cross-linking mass spectrometry provides evidence for coexisting respiratory supercomplexes
Source: Mol Cell Proteomics. 2017 Dec 8;17(2):216–32. doi: 10.1074/mcp.RA117.000470 (PMC5795388; doi:10.1074/mcp.RA117.000470)
Supplement: Supplemental Data [file supp_RA117.000470_133922_0_supp_23978_fzffwf.zip › spectra_annotation/mito_spectra_annotation/290-1-4-1-10-1.pdf]

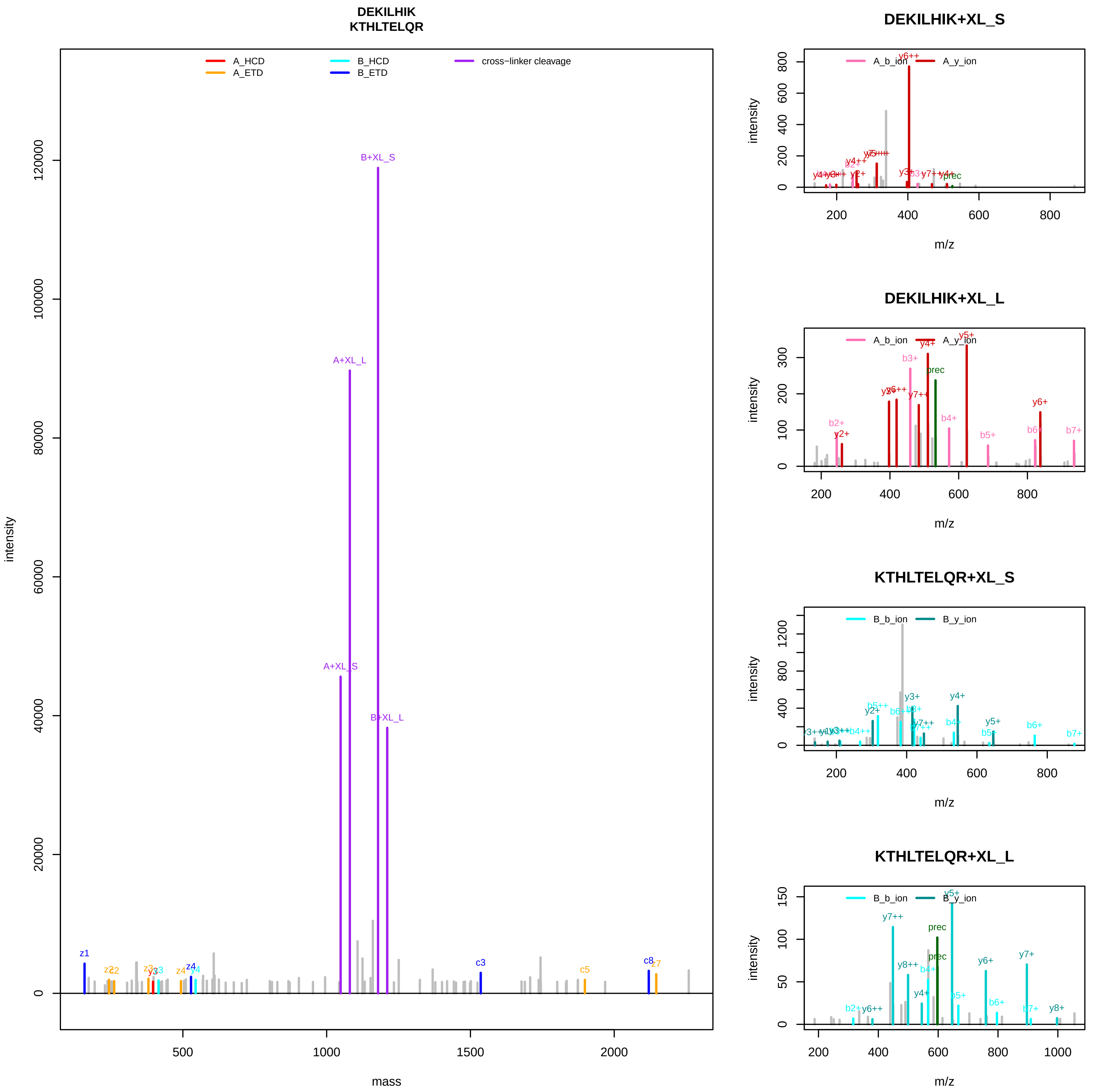

DEKILHIK
KTHLTELQR
A_HCD
A_ETD
B_HCD
B_ETD
cross−linker cleavage
B+XL_S
A+XL_L
A+XL_S
B+XL_L
z1
c3
c5
c8
z7
z4
y4
c2
z3
y3
intensity
mass
0
20000
40000
60000
80000
100000
120000
500
1000
1500
2000
DEKILHIK+XL_S
A_b_ion
A_y_ion
y6++
y2+
y3+
prec
m/z
DEKILHIK+XL_L
y5+
y4+
b3+
prec
y7++
b2+
y2+
b4+
b5+
y6+
b7+
KTHLTELQR+XL_S
B_b_ion
B_y_ion
y3+
y4+
y2+
b5++
y5+
b4+
b5+
b6+
b7+
KTHLTELQR+XL_L
y5+
y7++
prec
y8++
y6+
y7+
b2+
y6++
y4+
b5+
b6+
y8+
200
400
600
800
1000
